# Nutrition, dietary interventions and prostate cancer: the latest evidence

**DOI:** 10.1186/s12916-014-0234-y

**Published:** 2015-01-08

**Authors:** Pao-Hwa Lin, William Aronson, Stephen J Freedland

**Affiliations:** 1 Department of Medicine, Division of Nephrology, Duke University Medical Center, Box 3487, Durham, NC 27710 USA; 2 Urology Section, Department of Surgery, Veterans Affairs Greater Los Angeles Healthcare System, Los Angeles, CA USA; 3 Department of Urology, UCLA School of Medicine, Los Angeles, CA USA; 4 Urology Section, Department of Surgery, Durham Veterans Affairs Medical Center, Division of Urology, Durham, NC USA; 5 Duke Prostate Center, Departments of Surgery and Pathology, Duke University Medical Center, Durham, NC USA

**Keywords:** Diet, Prostate cancer, Nutrients, Dietary pattern, Lifestyle, Prevention, Treatment, Nutrition, Dietary intervention, Review

## Abstract

Prostate cancer (PCa) remains a leading cause of mortality in US men and the prevalence continues to rise world-wide especially in countries where men consume a ‘Western-style’ diet. Epidemiologic, preclinical and clinical studies suggest a potential role for dietary intake on the incidence and progression of PCa. 'This minireview provides an overview of recent published literature with regard to nutrients, dietary factors, dietary patterns and PCa incidence and progression. Low carbohydrates intake, soy protein, omega-3 (w-3) fat, green teas, tomatoes and tomato products and zyflamend showed promise in reducing PCa risk or progression. A higher saturated fat intake and a higher β-carotene status may increase risk. A ‘U’ shape relationship may exist between folate, vitamin C, vitamin D and calcium with PCa risk. Despite the inconsistent and inconclusive findings, the potential for a role of dietary intake for the prevention and treatment of PCa is promising. The combination of all the beneficial factors for PCa risk reduction in a healthy dietary pattern may be the best dietary advice. This pattern includes rich fruits and vegetables, reduced refined carbohydrates, total and saturated fats, and reduced cooked meats. Further carefully designed prospective trials are warranted.

## Introduction

Prostate cancer (PCa) is the second most common cancer in men, with nearly a million new cases diagnosed worldwide per year [1], with approximately a six-fold higher incidence in Western than in non-Western countries. Diet, lifestyle, environmental and genetic factors are hypothesized to play a role in these differences. This review focuses on the latest evidence of the potential role of dietary factors on PCa and includes epidemiologic and clinical trial evidence for the impact of protein, fat, carbohydrate, fiber, phytochemicals, other food components, whole foods and dietary patterns on PCa incidence, development and/or progression.

Data from meta-analyses or well-designed randomized trials and prospective studies are emphasized in this review. It should be noted that studies of dietary intake or nutrition and cancer are often subject to various limitations and thus complicate interpretation of results. For example, when a study is designed to examine the effect of the amount of fat intake, alteration in fat intake inevitably will change intake of protein and/or carbohydrate, and may change the intake of other nutrients as well. As a result, it is difficult to attribute the effect to change in fat intake alone. In addition, the impact of macronutrients potentially involves aspects of both absolute quantity and the type of macronutrients consumed. Both aspects may potentially affect cancer initiation and/or development independently, but they are not always distinguishable in research designs. Though this topic was recently reviewed [2], given the extensive new literature on the topic, an updated review is presented herein and a summary table is provided for a quick reference (Table 1).

**Table 1:** **Summary of nutrients and food factors with prostate cancer**

**Nutrient or food factor and references**	**Preclinical study**	**Epidemiological study**	**Clinical study**	**Summary**
Carbohydrate [3-7]	Low carbohydrate slowed tumor growth.	Rare	On-going	Potential, awaits evidence from RCT
Protein [23-46]	Soy protein slowed tumor progression. Genistein inhibited PCa cell detachment, invasion, and metastasis.	Mixed results in total protein, dairy, soy intake.	Supplement of geneistein reduced PCa progression.	Soy products showed potential benefit, need more RCT to confirm.
Fat [47-84]	Low fat reduced PCa risk, high fat increased risk.	Mixed results. Saturated fat may increase and plant fat decrease PCa risk. W-3 PUFA may decrease risk.	Low fat plus w-3 PUFA reduced PCa proliferation and CCP.	Further research needed to clarify role of amount and type of total fat and fatty acids.
Vitamin A [85-90]	NA	Higher serum β-carotene associated with higher PCa risk.	β-carotene supplement increased PCa risk.	Supplement not advised.
Folate [91-98]	Folate depletion reduced tumor growth.	Higher circulation folate associated with higher PCa risk or lower PSA.	Supplement folate had no effect on PCa risk.	Potential dual role of folate in prostate carcinogenesis, needs further examination.
Vitamin C [89]	May slow tumor growth *in vitro* and *in vivo*.	Rare	Rare. One study showed no effect.	May act both as pro-oxidant and antioxidant. Needs clarification.
Vitamin D [100-114]	May slow PCa progression.	Serum vitamin D associated with a higher or lower risk.	No impact of vitamin D supplement on PSA or PCa risk.	A ‘U’ shaped relationship may exist between vitamin D status and PCa.
Vitamin E [115-125]	May slow PCa tumor growth.	Some show no association between vitamin E supplement and PCa risk.	400 IU supplement had no effect or increased PCa risk, but 75 IU supplement lowered risk.	Weak evidence of benefit, further research should consider dosage also.
Vitamin K [126,127]	Anti-tumor, chemo and potential radiosensitizers.	Inverse relationship between vitamin K and PCa incidence.	NA	Inadequate evidence
Calcium [128-130]	Rare	Calcium intake increased or decreased PCa risk.	A ‘U’ shaped relationship may exist.	Further research is needed to clarify if any association exists.
Selenium [123,125,131,132]	Inhibit angiogenesis, proliferation, inducing apoptosis.	Toenail selenium associated with reduced advanced PCa risk.	Selenium supplement had no effect for PCa chemoprevention, or increased high grade PCa risk among men with high selenium status.	Conflicting results, more research is needed.
Silibinin [133-135]	Inhibit PCa growth via EGFR, IGF-1R, NF-kB, TGFβ2, and CAF-like markers.	NA	NA	Potential as chemopreventive agent, awaits further research.
Curcumin [136,137]	Inhibited proinflammatory NF-B, induced apoptosis, slowed PCa growth.	NA	NA	Potential to slow PCa growth, awaits further research.
Pomegranate [137-141]	Inhibited PCa proliferation, angiogenesis.		Pomegranate juice increased PSA doubling time in one trial, but no controls included. Another trial showed no impact.	Weak evidence of benefit.
Green tea [131,137,142-145]	Inhibited PCa growth, induced apoptosis, decreased inflammation.	NA	Green tea catechin or EGCG supplement reduced PCa incidence or PSA.	Some evidence of benefit, more research needed.
Resveratrol [137,146-151]	Inhibited PCa growth in some but not all studies.	NA	NA	Potential, awaits further study.
Zyflamend [152-157]	Reduced PCa progression.	NA	Reduced risk among those with high grade PCa.	Potential to slow PCa growth.
Fruits and vegetables [158-163]	Allium vegetable reduced PCa risk.	Inverse relationship between total fruit and vegetable intake and PCa risk.	Supplement of pomegranate, green tea, broccoli, turmeric reduced PSA rise.	Moderate evidence, consistent with current dietary guideline to encourage rich intake.
Tomatoes and products [131,164-176]	Lycopene slowed PCa growth, progression.	Higher lycopene intake or serum level associated with lower PCa risk in some studies.	Supplement lycopene lowered PSA, PCa symptoms in some studies.	Moderate evidence, needs large RCT to confirm.
Coffee [177-183]	NA	Inverse between coffee consumption and PCa risk in some studies but not all.	NA	Potential benefit.
Dietary pattern [70,184-191]	NA	High HEI associated with lower PCa risk. Mediterranean diet may prevent PCa. Western diet associated with higher PCa risk and Asian diet opposite.	NA	Promising. RCT needed.

CAF, cancer-associated fibroblasts; CCP, cell cycle progression; EGCG, epigallocatechin gallate; EGFR, epidermal growth factor receptor; HEI, Healthy Eating Index; IGF-1R, IGF1 receptor; NF-kB, nuclear factor kappa B; PCa, prostate cancer; PA, prostate specific antigen; PUFA, polyunsaturated fatty acid; RCT, randomized controlled trial; TGFβ2, transforming growth factor β2.

## Nutrients

### Carbohydrates

Given the hypothesis that insulin is a growth factor for PCa, it has been hypothesized that reducing carbohydrates and thus lowering serum insulin may slow PCa growth [3]. Indeed, in animal models, either a no-carbohydrate ketogenic diet (NCKD) [4,5] or a low-carbohydrate diet (20% kcal as carbohydrate) has favorable effects on slowing prostate tumor growth [6,7]. In human studies, one study found that high intake of refined carbohydrates was associated with increased risk of PCa [7]. In addition to the amount of carbohydrates, type of carbohydrates may impact on PCa but research has been inconclusive.

The potential to reduce PCa risk and progression via impacting carbohydrate metabolism is actively being investigated with Metformin. Metformin reduced PCa cell proliferation and delayed progression *in vitro* and *in vivo*, respectively [8-10] and reduced incident risk and mortality in humans [11-13]. Two single arm clinical trials also showed a positive effect of metformin in affecting markers of PCa proliferation and progression [14,15]. However, other retrospective cohort studies have not supported an effect of metformin on recurrence or incident risk of PCa [16-22].

Despite the potential for reducing either total or simple carbohydrates in benefiting PCa control, evidence is lacking from randomized controlled trials (RCT). Two randomized trials are on-going examining the impact of a low-carbohydrate diet (approximately 5% kcal) on the PSA doubling time among PCa patients post radical prostatectomy (NCT01763944) and on glycemic response among patients initiating androgen deprivation therapy (ADT) (NCT00932672 ). Findings from these trials will shed light on the effect of carbohydrate intake on markers of PCa progression and the role of reduced carbohydrate intake on offsetting the side effects of ADT.

### Protein

The ideal level of protein intake for optimal overall health or prostate health is unclear. Despite the popularity of low carbohydrate diets that are high in protein, recent human studies reported that low protein intake was associated with lower risk for cancer and overall mortality among men 65 and younger. Among men older than 65, low protein intake was associated with a higher risk for cancer and overall mortality [23]. In animal models the ratio between protein and carbohydrate impacted on cardiometabolic health, aging and longevity [24]. The role of dietary protein and the protein to carbohydrate ratio on PCa development and progression requires further study.

#### Animal-based proteins

Studying protein intake, like all aspects of nutritional science, can be challenging. For example, animal meat, which is a source of protein in Western diets, is composed not only of protein, but also of fat, cholesterol, minerals and other nutrients. The amount of these nutrients including fatty acids may vary from one animal meat to the other. Previous studies in human have shown that consumption of skinless poultry, which is lower in cholesterol and saturated fat than many red meats, was not associated with the recurrence or progression of PCa [25]. However, consumption of baked poultry was inversely associated with advanced PCa [26,27], while cooked red meat was associated with increased advanced PCa risk [26,27]. Thus, how the food is prepared may modify its impact on PCa risk and progression. Overall, fish consumption may be associated with reduced PCa mortality, but high temperature cooked fish may contribute to PCa carcinogenesis [28]. Thus, it may be advisable to consume fish regularly but cooking temperature should be kept moderate.

#### Dairy-based protein

Another common protein source is dairy products, such as milk, cheese and yogurt. Previous studies have shown that dairy increased overall PCa risk but not with aggressive or lethal PCa [29,30]. In addition, both whole milk and low-fat milk consumption were reported to either promote or delay PCa progression [29,31]. In the Physicians Health follow up cohort with 21,660 men, total dairy consumption was found to be associated with increased PCa incidence [32]. In particular, low fat or skim milk increased low grade PCa, whereas whole milk increased fatal PCa risk. Though the exact component(s) of dairy products driving these associations is unknown, the high concentrations of saturated fat and calcium may be involved. A cross-sectional study of 1798 men showed that dairy protein was positively associated with serum IGF-1 [33] levels which may stimulate initiation or progression of PCa. Thus, further research is needed to clarify the relationship between dairy intake and PCa. There is insufficient data to provide recommendations specifically related to dairy or dairy protein and PCa risk or progression.

#### Plant-based proteins

Soy and soy-based products are rich in protein and phytoestrogens that may facilitate PCa prevention, but its role on PCa is unclear. In a study in mice, intake of soy products was associated with decreased hepatic aromatase, 5α-reductase, expression of androgen receptor and its regulated genes, FOXA1, urogenital tract weight and PCa tumor progression [34]. A recent randomized trial of 177 men with high-risk disease after radical prostatectomy found that soy protein supplementation for two years had no effect on risk of PCa recurrence [35]. Although epidemiological and pre-clinical studies [36,37] support a potential role for soy/soy isoflavones in PCa risk reduction or progression, a meta-analysis did not find significant impact of soy intake in PSA levels, sex hormone-binding globulin, testosterone, free testosterone, estradiol or dihydrotestosterone [38]. Another RCT in patients before prostatectomy also did not find any effect of soy isoflavone supplement up to six weeks on PSA, serum total testosterone, free testosterone, total estrogen, estradiol or total cholesterol [39]. Since most RCTs conducted have been small and of short duration, further examination is needed.

Many studies have continued to examine the primary isoflavone in soy, genistein, and its effect on PCa. The potential for genistein to inihibit PCa cell detachment, invasion and metastasis is reported [40]. Genistein may modify glucose update and glucose transporter (GLUT) expression in PCa cells [41], or exert its anti-tumor effect by down regulating several microRNAs [42]. Studies using tumor cells and animal models suggest genistein may compete with and block endogenous estrogens from binding to the estrogen receptor, thereby inhibiting cellular proliferation, growth, and inducing differentiation and, specifically, genistein may inhibit cell detachment, protease production, cell invasion and thus prevent metastasis [36,40,43]. However, neither plasma nor urinary genistein levels were associated with PCa risk in case control studies [44,45]. In a phase 2 placebo-controlled RCT with 47 men, supplementation of 30 mg genistein for three to six weeks significantly reduced androgen-related markers of PCa progression [46]. In addition, genistein may be beneficial in improving cabazitaxel chemotherapy in metastatic castration-resistant PCa [37]. Clinical studies are warranted to further examine the role of soy and soy isoflavones for PCa prevention or treatment. A definitive recommendation regarding protein intake for PCa prevention or treatment is not available yet.

### Fat

Research findings examining fat consumption with PCa risk or progression are conflicting. Both the total absolute intake [47] of dietary fat and the relative fatty acid composition may independently relate to PCa initiation and/or progression. While animal studies repeatedly show that reducing dietary fat intake slows tumor growth [48-50] and high fat diets, especially animal fat and corn oil increase PCa progression [51], human data are less consistent. Case–control studies and cohort studies have shown either no association between total fat consumption and PCa risk [52-55] or an inverse association between fat intake and PCa survival, particularly among men with localized PCa [47]. In addition, a cross-sectional study showed that fat intake expressed as percent of total calorie intake was positively associated with PSA levels in 13,594 men without PCa [56].

Given these conflicting data, it is possible that the *type* of fatty acid [56] rather than total amount may play an important role in PCa development and progression. A study found plasma saturated fatty acids to be positively associated with PCa risk in a prospective cohort of 14,514 men of the Melbourne Collaborative Cohort Study [57]. In addition, another study found that eating more plant-based fat was associated with reduced PCa risk [58]. These studies support the current dietary guideline of eating less animal-based fat and more plant-based fat.

The data regarding omega-6 (w-6) and omega-3 (w-3) polyunsaturated fatty acid (PUFA) consumption and PCa risk are also conflicting. While there are data to support a link between increased w-6 PUFA intake (mainly derived from corn oil) and risk of overall and high-grade PCa [57,59], not all data support such a link [60]. In fact, a greater polyunsaturated fat intake was associated with a lower all cause mortality among men with nonmetastatic PCa in the Health Professionals Follow-up study [58]. The postulated mechanism linking w-6 PUFAs and PCa risk is the conversion of arachidonic acid (w-6 PUFA) to eicosanoids (prostaglandin E-2, hydroxyeicosatetraenoic acids and epoxyeicosatrienoic acids) leading to inflammation and cellular growth [61]. Conversely, w-3 PUFAs, which are found primarily in cold water oily fish, may slow growth of PCa through a number of mechanisms [61-63]. In a study of 48 men with low risk PCa under active surveillance, repeat biopsy in six months showed that prostate tissue w-3 fatty acids, especially eicosapentaenoic acid (EPA), may protect against PCa progression [64]. *In vitro* and animal studies suggest that w-3 PUFAs induce anti-inflammatory, pro-apoptotic, anti-proliferative and anti-angiogenic pathways [65,66]. Moreover, a mouse study comparing various types of fat found that only the fish oil diet (that is, omega-3 based diet) slowed PCa growth relative to other dietary fats [67]. In regards to human data, a phase II randomized trial showed that a low-fat diet with w-3 supplementation four to six weeks prior to radical prostatectomy decreased PCa proliferation and cell cycle progression (CCP) score [62,68]. A low-fat fish oil diet resulted in decreased 15(S)-hydroxyeicosatetraenoic acid levels and lowered CCP score relative to a Western diet [69]. The potential benefits of omega-3 fatty acids from fish are supported by epidemiological literature showing that w-3 fatty acid intake was inversely associated with fatal PCa risk [70,71]. Despite the promise of omega-3 fatty acids, not all studies agree. Supplementing 2 g alpha-linolenic acid (ALA) per day for 40 months in 1,622 men with PSA <4 ng/ml did not change their PSA [72]. However, another study found that a high blood serum n-3 PUFA and docosapentaenoic acid (DPA) was associated with reduced total PCa risk while high serum EPA and docosahexaenoic acid (DHA) was possibly associated with increased high-grade PCa risk [73]. Further research is required to understand better the role of omega-3 PUFAs in PCa prevention or treatment.

### Cholesterol

Many pre-clinical studies have shown that the accumulation of cholesterol contributes to the progression of PCa [74-76]. It was suggested that a high cholesterol in circulation may be a risk factor for solid tumors, primarily through the upregulation of cholesterol synthesis, inflammatory pathways [77] and intratumoral steroidogenesis [78]. According to a recent study with 2,408 men scheduled for biopsy, serum cholesterol was independently associated with prediction of PCa risk [79]. Consistent with the cholesterol findings, usage of the cholesterol lowering drug statin post radical prostatectomy (RP) was significantly associated with reduced risk of biochemical recurrence in 1,146 radical prostatectomy patients [80]. Another study also showed that statins may reduce PCa risk by lowering progression [81]. Although the mechanism has not been established, more recent studies also showed that a low high-density lipoprotein (HDL) cholesterol level was associated with a higher risk for PCa and, thus, a higher HDL was protective [81-84]. These findings support the notion that a heart-healthy dietary intervention that lowers cholesterol may benefit prostate health also.

### Vitamins and minerals

Herein we will review the recent data on vitamins A, B complex, C, D, E, and K and selenium. In the two large clinical trials: the Carotene and Retinol Efficacy Trial (CARET; PCa was a secondary outcome) and the National Institutes of Health-American Association of Retired Persons (NIH-AARP) Diet and Health prospective cohort study, excessive multivitamin supplementation was associated with a *higher* risk of developing aggressive PCa, particularly among those taking individual β-carotene supplements [85,86]. Similarly, high serum β-carotene levels were associated with a higher risk for PCa among 997 Finnish men in the Kuopio Ischaemic Heart Disease Risk Factor cohort [87]. However, β-carotene supplement was not found to affect risk for lethal PCa during therapy [88], or in the Danish prospective cohort study of 26,856 men [89]. Circulating retinol also was not associated with PCa risk in a large case–control study [90]. Thus, the association between vitamin A and PCa is still unclear.

Preclinical evidence suggests folate depletion may slow tumor growth, while supplementation has no effect on growth or progression, but may directly lead to epigenetic changes via increases in DNA methylation [91]. Two meta-analyses also showed that circulating folate levels were positively associated with an increased risk of PCa [92,93], while dietary or supplemental folate had no effect on PCa risk [94] in a cohort study with 58,279 men in the Netherlands [95] and a case–control study in Italy and Switzerland [96]. In fact, one study of a cohort of men undergoing radical prostatectomy at several Veterans Administration facilities across the US even showed that higher serum folate levels were associated with lower PSA and, thus, lower risk for biochemical failure [97]. Another study using data from the 2007 to 2010 National Health and Nutrition Examination Survey showed that a higher folate status may be protective against elevated PSA levels among 3,293 men, 40-years old and older, without diagnosed PCa [98]. It was suggested that folate may play a dual role in prostate carcinogenesis and, thus, the complex relationship between folate and PCa awaits further investigation [99].

Despite the potential role of vitamin C (ascorbic acid) as an antioxidant in anticancer therapy, trials examining dietary intake or supplementation of vitamin C are few. A RCT showed no effect of vitamin C intake on PCa risk [89]. Furthermore, vitamin C at high doses may act more as a pro-oxidant than antioxidant, complicating the research design and interpretation.

The primary active form of vitamin D, 1,25 dihydroxyvitamin D3 (calcitriol) aids in proper bone formation, induces differentiation of some immune cells, and inhibits pro-tumor pathways, such as proliferation and angiogenesis, and has been suggested to benefit PCa risk [100]; however, findings continue to be inconclusive. More recent studies found that increased serum vitamin D levels were associated with *decreased* PCa risk [101,102]. Further, supplementing vitamin D may slow PCa progression or induce apoptosis in PCa cells [103-105]. Other studies, however, reported either no impact of vitamin D supplement on PSA [106] or no effect of vitamin D status on PCa risk [107,108]. Some studies contrarily reported that a lower vitamin D status was associated with a lower PCa risk in older men [109], or a higher serum vitamin D was associated with a higher PCa risk [110,111]. A study even suggested that a ‘U’ shaped relationship may exist between vitamin D status and PCa and the optimal range of circulating vitamin D for PCa prevention may be narrow [112]. This is consistent with the findings for other nutrients that a greater intake of a favorable nutrient may not always be better.

A recent study showed that the association between vitamin D and PCa was modulated by vitamin D-binding protein [113] which may have partially explained the previous inconsistent findings. Further, a meta-analysis investigating the association between Vitamin D receptor (VDR) polymorphisms (BsmI and FokI) and PCa risk reported no relationship with PCa risk [114]. Thus, the role of vitamin D in PCa remains unclear.

In a large randomized trial with a total of 14,641 US male physicians ≥50-years old, participants randomly received 400 IU of vitamin E every other day for an overall mean of 10.3 (13.8) years. Vitamin E supplementation had no immediate or long-term effects on the risk of total cancers or PCa [115]. However, a moderate dose of vitamin E supplement (50 mg or about 75 IU) resulted in lower PCa risk among 29,133 Finnish male smokers [116]. Multiple preclinical studies suggest vitamin E slows tumor growth, partly due to inhibiting DNA synthesis and inducing apoptotic pathways [117]. Unfortunately, human studies have been less than supportive. Two observational studies (the Cancer Prevention Study II Nutrition Cohort and the NIH-AARP Diet and Health Study) both showed no association between vitamin E supplementation and PCa risk [118,119]. However, a higher serum α-tocopherol but not the γ-tocopherol level was associated with decreased risk of PCa [120,121] and the association may be modified by genetic variations in vitamin E related genes [122]. On the contrary, a prospective randomized trial, the Selenium and Vitamin E Cancer Prevention Trial (SELECT), showed vitamin E supplementation significantly *increased* PCa risk [123] and that a higher plasma α-tocopherol level may interact with selenium supplements to increase high grade PCa risk [124]. This finding is consistent with a case-cohort study of 1,739 cases and 3,117 controls that showed vitamin E increased PCa risk among those with low selenium status but not those with high selenium status [125]. Thus, more research is needed to examine the association between vitamin E and PCa and the dose effect and interaction with other nutrients should be considered.

Vitamin K has been hypothesized to help prevent PCa by reducing bioavailable calcium. Preclinical studies show the combination of vitamins C and K have potent anti-tumor activity *in vitro* and act as chemo- and radiosensitizers *in vivo* [126]. To date, few studies have investigated this, although one study using the European Prospective Investigation into Cancer and Nutrition (EPIC)-Heidelberg cohort found an inverse relationship between vitamin K (as menaquinones) intake and PCa incidence [127].

Little to no preclinical studies have been conducted to examine the role of calcium with PCa. Retrospective and meta-analyses suggest increased or reduced PCa risk with increased calcium intake, while others suggest no association [128,129]. Another study suggests a ‘U’-shaped association, where very low calcium levels *or* supplementation are both associated with PCa [130].

Selenium, on the other hand, has been hypothesized to prevent PCa. While *in vitro* studies suggested that selenium inhibited angiogenesis and proliferation while inducing apoptosis [131], results from SELECT showed no benefit of selenium alone or in combination with vitamin E for PCa chemoprevention [123]. Further, selenium supplementation did not benefit men with low selenium status but increased the risk of high-grade PCa among men with high selenium status in a randomly selected cohort of 1,739 cases with high-grade (Gleason 7–10) PCa and 3,117 controls [125]. A prospective Netherlands Cohort Study, which included 58,279 men, 55- to 69-years old, also showed that toenail selenium was associated with a reduced risk of advanced PCa [132]. Further research is needed to clarify the role of selenium with PCa.

## Phytochemicals

Along with vitamins and minerals [2], plants contain phytochemicals with potential anti-cancer effects. Typically not considered essential compounds, phytochemicals have antioxidant and anti-inflammatory properties.

Silibinin is a polyphenolic flavonoid found in the seeds of milk thistle. It has been shown *in vitro* and *in vivo* to inhihit PCa growth by targeting epidermal growth factor receptor (EGFR), IGF-1 receptor (IGF-1R), and nuclear factor-kappa B (NF-kB) pathways [133,134]. A recent study showed that silibinin may be useful in PCa prevention by inhibiting TGFβ2 expression and cancer-associated fibroblast (CAF)-like biomarkers in the human prostate stromal cells [135]. Thus, silibinin is a promising candidate as a PCa chemopreventive agent that awaits further research.

Curcumin is used as food additive in Asia and as an herbal medicine for inflammation [136]. *In vitro*, curcumin inhibits the pro-inflammatory protein NF-κB while inducing apoptosis through increased expression of pro-apoptotic genes [137]. *In vivo*, curcumin slows PCa growth in mice while sensitizing tumors to chemo- and radiotherapies [136]; however, no human trial has examined its impact on PCa.

### Pomegranate

The peel and fruit of pomegranates and walnuts are rich in ellagitannins (punicalagins). These phytochemicals are readily metabolized to the active form ellagic acid by gut flora [138]. Preclinical experiments show ellagitannins inhibit PCa proliferation and angiogenesis under hypoxic conditions and induce apoptosis [137,138]. In prospective trials in men with a rising PSA after primary treatment, pomegranate juice or POMx, a commercially available pomegranate extract, increased the PSA doubling time relative to baseline [139,140], although no trials included a placebo group. Results are pending from a prospective placebo RCT using pomegranate extract in men with a rising PSA. However, in a placebo controlled trial, two pills of POMx daily for up to four weeks prior to radical prostatectomy had no impact on tumor pathology or oxidative stress or any other tumor measures [141].

### Green tea

Green tea contains a number of antioxidant polyphenols including catechins, such as epigallocatechin gallate (EGCG), epigallocatechin (EGC), (−)-epicatechin-3-gallate (ECG) and (−)-epicatechin. Preclinical studies suggest EGCG inhibits PCa growth, induces intrinsic and extrinsic apoptotic pathways and decreases inflammation by inhibiting NFkB [137]. Furthermore, the antioxidant properties of EGCG are 25 to 100 times more potent than vitamins C and E [131]. In a prospective randomized preprostatectomy trial, men consuming brewed green tea prior to surgery had increased levels of green tea polyphenols in their prostate tissue [142]. In a small proof-of-principle trial with 60 men, daily supplementation of 600 mg green tea catechin extract reduced PCa incidence by 90% (3% versus 30% in the placebo group) [143]. Another small trial also showed that EGCG supplement resulted in a significant reduction in PSA, hepatocyte growth factor and vascular endothelial growth factor in men with PCa [144]. These studies suggest green tea polyphenols may lower PCa incidence and reduce PCa progression but more research is needed to confirm and clarify its mechanism [137,143,145].

### Resveratrol

While most *in vitro* studies suggest resveratrol inhibits PCa growth [146-148], resveratrol suppresses tumor growth in some [137] but not all animal models [149], possibly due to limited bioavailability [150,151]. To date, there are no clinical trials investigating the preventive or therapeutic effects of resveratrol on PCa.

### Zyflamend

Zyflamend is an anti-inflammatory mixture of herbs that has been shown to reduce PCa progression by lowering the expression of markers including pAKT, PSA, histone deacetylases and androgen receptor in animal models and PCa cell line [152-154]. Despite its anti-cancer potential [155], very few studies have been conducted in humans [156,157]. In an open-label Phase I trial of 23 patients with high-grade prostatic intraepithelial neoplasia, Zyflamend alone or in conjunction with other dietary supplements for 18 months reduced the risk for developing PCa [156]. More RCTs in humans are needed to confirm the efficacy and clinical application of this herbal supplement.

## Other whole foods

### Fruits and vegetables

Fruits and vegetables are rich sources of vitamins, minerals and phytochemicals. Several epidemiologic studies found inverse relationships between total fruit and vegetable intake [158], and cruciferous vegetable intake and PCa risk [159,160]. Allium vegetables, such as garlic, leeks, chives, and shallots, contain multiple sulfurous phytochemicals that were suggested to enhance the immune system, inhibit cell growth, modulate expression of androgen-responsive genes and induce apoptosis [161]. Although the number of published studies is limited, both preclinical and epidemiologic data suggest allium vegetable intake may be protective against PCa, particularly localized disease [162]. A randomized trial with 199 men also found that a blend supplement of pomegranate, green tea, broccoli and turmeric significantly reduced the rate of rise in PSA in men with PCa [163].

### Tomatoes and tomato products

A number of studies have examined the association between tomatoes and tomato products with PCa but the findings are inconclusive. The antioxidant lycopene, which is rich in tomatoes, has also been studied specifically for its impact on PCa. *In vitro*, lycopene halts the cell cycle in several PCa cell lines and decreases IGF-1 signaling by inducing IGF-1 binding proteins [131]. While some animal studies found lycopene specifically slows PCa growth [164] or reduces PCa epithelial cells at stages of initiation, promotion and progression [165], two studies found conflicting findings between tomato paste and lycopene [166,167]. Prospective human studies found higher lycopene consumption [168,169] or higher serum levels were associated with lower PCa risk [170], but others have not [171,172]. Prostatic lycopene concentration below a 1 ng/mg threshold was associated with PCa at six-month follow-up biopsy (*P* = 0.003) [173]. Two short-term preprostatectomy trials using tomato sauce or lycopene supplementation demonstrated lycopene uptake in prostate tissue and antioxidant and potential anticancer effects [174,175]. While several clinical trials suggested an inverse relationship between lycopene supplementation, PSA levels and decreases in cancer-related symptoms [171,176], no large-scale randomized trials have tested the role of lycopene or tomato products on PCa prevention or treatment.

### Coffee

Coffee contains caffeine and several unidentified phenolic compounds that may serve as antioxidants. Epidemiological studies suggest an inverse relationship between coffee consumption and PCa risk, mainly for advanced or lethal stage disease, and the findings were independent of caffeine content [177,178]. Although several epidemiological studies [179-182] found no association between coffee consumption and PCa risk, a recent meta-analysis of prospective studies concluded that coffee consumption may reduce PCa risk [183]. The potential mechanism(s) and pathway(s) involved are unknown but may include antioxidant, anti-inflammatory effects, glucose and insulin metabolism, and potential impact on IGF-I and circulating sex hormones.

## Dietary patterns

Even though many single nutrients or food factors have been examined for their impact or association with PCa risk or progression, the results have largely been inconclusive. A potential reason for the inconsistency is the fact that the impact of single nutrient or food factor may be too small to be detected. In addition, nutrients naturally existing in foods often are highly correlated and may interact with each other and, thus, affect the impact on PCa. Thus, dietary pattern analysis has received an increasing interest but research has been limited and the existing results have been inconclusive. In a cohort of 293,464 men, a high dietary quality, as indicated by the Healthy Eating Index (HEI) score, was associated with a lower risk of total PCa risk [70]. The Mediterranean diet, which is high in vegetables, olive oil, complex carbohydrates, lean meats and antioxidants, is consistently recommended to patients for prevention of cardiovascular disease and obesity [184], and may show promise in PCa prevention [185]. Fish and omega-3 fatty acid consumption in the Mediterranean pattern were significantly and inversely associated with fatal PCa risk. In addition, adherence to the Mediterranean diet after diagnosis of non-metastatic PCa was associated with lower overall mortality [186]. Whereas, a Western pattern with high intakes of red meats, processed meats, fried fish, chips, high-fat milk and white bread, was associated with a higher risk for PCa [187].

Furthermore, Asian countries with high consumption of omega-3 PUFAs, soy and green tea-based phytochemicals, have lower PCa incidences versus countries consuming a ‘Western-style’ diet [188]. However, not all studies [189-191] supported an association between certain dietary pattern and risk of PCa. It is possible that the methodology used in identifying dietary patterns may not have captured all the dietary factors associated with PCa risk. Alternatively, each dietary pattern may contain both beneficial and harmful components resulting in an overall null association. More research is needed to continue searching for dietary patterns that combine most of the beneficial nutrients/food factors for PCa and limit most of the negative nutrients/food factors.

## Future direction for clinical trials

Based on the multitude of epidemiologic, preclinical and clinical trials described in this review, dietary interventions for the prevention and treatment of PCa hold great promise. In addition, several dietary factors and vitamins/supplements may be associated with PCa risk and/or progression of disease. Prospective randomized trials are clearly indicated to identify specific nutrients or combination therapies for the prevention and treatment of PCa.

Recently, active surveillance (AS) has emerged as a viable option for men with lower risk PCa. Men on AS are motivated to adhere to diet and lifestyle modifications [192], making this subset a good target for dietary intervention and quality of life trials [193]. PCa survivors who are more active and report ‘healthy’ eating habits (that is, consuming low-fat, low-refined carbohydrate diets rich in fruits and vegetables) have better overall quality of life versus their inactive, unhealthy counterparts [194]. Thus, more randomized trials are warranted to determine the overall long-term effects of dietary intervention in this population. Specifically, key questions to address in future trials are: 1) Can dietary interventions delay the need for treatment in men on AS; 2) Can dietary interventions prevent recurrence for men after treatment; 3) Can dietary interventions delay progression among men with recurrent disease and, thus, delay the need for hormonal therapy; 4) Can dietary interventions reduce the side effects of PCa treatments including hormonal therapy and newer targeted therapies; and 5) Is there any role for dietary interventions alone or combined with targeted therapies in men on hormonal therapy to prevent castrate-resistance or after the emergence of castrate resistance disease? Because increasing evidence shows that metabolic abnormalities increase risk for PCa, lifestyle intervention that improves metabolic profile is a win-win option for PCa prevention and treatment [195,196].

## Conclusions

Future research is required to determine the ideal diet for PCa prevention or treatment. However, several dietary factors and some dietary patterns hold promise in reducing PCa risk or progression and are consistent with current dietary guidelines for Americans [197]. For counseling patients on diet for primary and secondary PCa prevention, many believe ‘heart healthy equals prostate healthy.’ Thus, given the current inconclusive results, the best dietary advice for PCa prevention or management seems to include: increasing fruits and vegetables, replacing refined carbohydrates with whole grains, reducing total and saturated fat, reducing overcooked meats and consuming a moderate amount of calories or reducing carbohydrates with a primary goal of obtaining and maintaining a healthy body weight.
